# Unbiased curriculum learning enhanced global-local graph neural network for protein thermodynamic stability prediction

**DOI:** 10.1093/bioinformatics/btad589

**Published:** 2023-09-22

**Authors:** Haifan Gong, Yumeng Zhang, Chenhe Dong, Yue Wang, Guanqi Chen, Bilin Liang, Haofeng Li, Lanxuan Liu, Jie Xu, Guanbin Li

**Affiliations:** Shanghai Artificial Intelligence Laboratory, Shanghai 200000, China; School of Computer Science and Engineering, Sun Yat-sen University, Guangzhou 510000, China; SRIBD, Chinese University of Hong Kong (Shenzhen), Shenzhen 518000, China; Shanghai Jiao Tong University, Shanghai 200000, China; School of Computer Science and Engineering, Sun Yat-sen University, Guangzhou 510000, China; Qilu Hospital, Shandong University, Shandong 250000, China; School of Computer Science and Engineering, Sun Yat-sen University, Guangzhou 510000, China; Shanghai Artificial Intelligence Laboratory, Shanghai 200000, China; SRIBD, Chinese University of Hong Kong (Shenzhen), Shenzhen 518000, China; Shanghai Artificial Intelligence Laboratory, Shanghai 200000, China; Shanghai Artificial Intelligence Laboratory, Shanghai 200000, China; School of Computer Science and Engineering, Sun Yat-sen University, Guangzhou 510000, China

## Abstract

**Motivation:**

Proteins play crucial roles in biological processes, with their functions being closely tied to thermodynamic stability. However, measuring stability changes upon point mutations of amino acid residues using physical methods can be time-consuming. In recent years, several computational methods for protein thermodynamic stability prediction (PTSP) based on deep learning have emerged. Nevertheless, these approaches either overlook the natural topology of protein structures or neglect the inherent noisy samples resulting from theoretical calculation or experimental errors.

**Results:**

We propose a novel Global-Local Graph Neural Network powered by Unbiased Curriculum Learning for the PTSP task. Our method first builds a Siamese graph neural network to extract protein features before and after mutation. Since the graph’s topological changes stem from local node mutations, we design a local feature transformation module to make the model focus on the mutated site. To address model bias caused by noisy samples, which represent unavoidable errors from physical experiments, we introduce an unbiased curriculum learning method. This approach effectively identifies and re-weights noisy samples during the training process. Extensive experiments demonstrate that our proposed method outperforms advanced protein stability prediction methods, and surpasses state-of-the-art learning methods for regression prediction tasks.

**Availability and implementation:**

All code and data is available at https://github.com/haifangong/UCL-GLGNN.

## 1 Introduction

Proteins play an essential role in most biological processes, and their functions are realized through the dynamic structures (Frauenfelder *et al.* 1988, Li *et al.* 2022). Gaining insights into protein functions through the dynamic changes of their attributes (e.g. three-dimensional structure, thermodynamic stability) can help us better understand the fundamentals of life (Park *et al.* 2004). For example, certain diseases result from a single amino acid residue alteration, leading to a significant difference in protein thermodynamic stability that is closely related to the disease’s molecular mechanism (Hartl 2017). Recent years have witnessed the great success of the protein 3D structure prediction based on deep learning (Jumper *et al.* 2021), which accelerates the traditional folding structural prediction task from months to hours. Similarly, estimating the change of protein thermodynamic stability upon amino acid mutations using conventional physical approaches (Marabotti *et al.* 2021) is time-consuming and laborious. Therefore, accurate computational approaches for protein thermodynamic stability prediction (PTSP) are needed, which will contribute to research on mutation-induced diseases and precision medicine.

PTSP aims to quantitatively predict the change in protein thermodynamic stability, denoted as ΔΔG (Stefl *et al.* 2013, Pancotti *et al.* 2022), representing the difference between Gibbs free energies (ΔG). The Gibbs free energy ΔG is used to estimate the stability change of a protein from unfolding state to folding state. When a mutation occurs in an amino acid, it will disrupt the interaction network of amino acid residues, leading to changes in thermodynamic stability. For the folding protein without mutation, we refer it as a wide-type with Gibbs free energy change $\Delta G_{w}$. Conversely, the folding protein with amino acid mutation is called mutant structure with Gibbs free energy change $\Delta G_{m}$. Thus, the difference between Gibbs free energy is obtained with the formulation $\Delta \Delta G=\Delta G_{m}-\Delta G_{w}$.

Researchers have made great efforts (Pancotti *et al.* 2022) in the field of thermodynamic stability and developed several deep learning-based approaches for ΔΔG prediction (Pandurangan *et al.* 2017, Pucci *et al.* 2018, Montanucci *et al.* 2019, Li *et al.* 2020, Benevenuta *et al.* 2021). Unlike the computationally demanding methods based on biophysical modelings such as molecular dynamics simulation, deep-learning-based methods that extract features from protein sequences and structure have entered the mainstream. However, there remain two crucial unsolved problems in the way to provide more reliable predictions of thermodynamic stability upon point mutations: (i) The above-mentioned works either ignore the natural topology of proteins nor neglect the importance of the mutated site, which is the essential cause of topological changes of mutant proteins. (ii) The noisy samples are unavoidable in the PTSP task as the ΔΔG obtained by the experimental values could be affected by the environment and human operation. However, the previous works have neglected the noisy samples for the PTSP task, which influences the model generalization ability and robustness.

To address the above-mentioned challenges, we propose a Global-Local Graph Neural Network enhanced with Unbiased Curriculum learning (GLGNN-UCL) to predict changes in protein thermodynamic stability. GLGNN-UCL represents proteins as graphs, with amino acids as nodes and residue interactions as edges. We first construct a Siamese graph attention network (GAT) (Veličković *et al.* 2018) to prediction ΔΔG based on the global feature, the satisfactory accuracy showing that the geometric information is quite important. Still, the single point mutation site’s information, responsible for alterations in residue interactions (i.e. graph topology) in proteins, is not well considered. To address this issue, we devise a local feature transformation flow to enhance the model’s ability to represent the local mutated site’s features.

More importantly, we propose a novel unbiased curriculum learning method to handle the inherent noisy samples in the PTSP task. We develop a simple yet effective hard sample selection function that automatically identifies noisy samples and adjusts their weights, preventing the model from being influenced by noise samples. Our approach demonstrates state-of-the-art performance on common benchmarks compared to other methods. The contributions of this work are:

- We propose a framework named GLGNN-UCL to predict the change of protein thermodynamic stability upon point mutation. GLGNN-UCL exploits a Siamese graph neural network to represent the structure of the protein before the mutation and after the mutation. Followed by the logic of the nature of amino acid mutations, we use the local node feature to enhance the global feature representation to boost the performance.
- We elaborate an unbiased curriculum learning approach to handle the intrinsic noisy samples in the thermodynamic stability prediction task, which could effectively distinguish and reweights the noisy samples thus avoiding the model from being affected by the noise.
- We contribute a benchmark for PTST task based on graph structure, which includes a training-validation set with 2548 samples and 2 independent test sets with 852 samples.
- Extensive experiments on our benchmark demonstrate that our GLGNN-UCL not only significantly exceeds the previous state-of-the-art methods for thermodynamic stability prediction but also outperforms methods that aim to handle the noisy samples for regression tasks.

## 2 Related work

### 2.1 Protein thermodynamic stability prediction

Several deep learning methods have been employed to predict the changes in thermodynamic stability. INPS (Fariselli *et al.* 2015) adopted SVM regression to learn the biological features from the protein sequences. DynaMUT2 (Rodrigues *et al.* 2021) use the random forest to predict the protein’s thermodynamic stability change based on the graph signatures features of molecular. SDM (Pandurangan *et al.* 2017) used a set of conformationally constrained substitution tables to calculate the difference in stability between the wild-type and mutant structure. PopMusicSym (Pucci *et al.* 2018) selected ANN to predict ΔΔG with statistical potentials and solvent accessibility of the modified residue. DDGun3D (Montanucci *et al.* 2019) provided an untrained method introducing anti-symmetric features based on evolutionary information. ThermoNet (Li *et al.* 2020) generated voxelized features according to the biophysical properties around the mutation site, and treated protein structures as if they were multi-channel 3D images. ACDC-NN (Benevenuta *et al.* (2021) built a Siamese neural network to extract the sequence and structural features from both the wild-type and mutant protein structure. However, these approaches ignore the topological information of residue interaction among the protein structure and the influence of noisy samples has not been discussed. KORPM (Hernández *et al.* 2023) proposed a simple residue-based orientational potential model uses three backbone atoms to predict the thermodynamic stability change upon mutation.

### 2.2 Curriculum learning and regression

Curriculum learning (CL) (Bengio *et al.* 2009) stems from the idea that learning from easy to hard could improve the generalization ability of the model. Various works have demonstrated the merit of the CL in computer vision (Gong *et al.* 2022) or natural language processing (Platanios *et al.* 2019). Recently, Wang *et al.* (2021) propose the CurGraph that aims at solving the graph classification task by estimating the complexity of the graph’s topology. However, there are only several works that focus on training the model for a regression task with the curriculum, as the regression task is different from the classification task according to Yang *et al.* (2021). Castells *et al.* (2020) embeds the CL into regression task by proposing the SuperLoss that automatically decreases the contribution of samples with a large loss.

Regression based on imbalanced data is a common issue in the real world, especially in the domain of bioinfomatics. However, most efforts are mainly based on SMOTE (Torgo *et al.* 2013). Yang *et al.* (2021) proposed a deep imbalance regression (DIR) framework to handle this issue by taking both label and feature distribution calibration into account. Nevertheless, the DIR is mainly designed for the task in the domain of computer vision and natural language processing, and does not take the distance between targets into account.

### 2.3 Graph neural networks

Graph Neural Networks (GNN) are powerful tools to model the non-Euclidean data. Inspired by the convolution operation in the imaging data, Graph Convolutional Network (Kipf and Welling 2017) (GCN) was proposed to handle graph data. GraphSAGE (Hamilton *et al.* 2017) extends the GCN based on the idea of inductive learning. Graph Attention Network (Veličković *et al.* 2018) (GAT) learns a graph feature transformation with the masked self-attention mechanism. Graph Isomorphism Network (GIN) (Xu *et al.* 2019) provides a theoretical foundation for the expressive power of GNNs and the design of a powerful GNN.

Numerous studies have applied graph neural networks (GNNs) to biological problems, which includes protein design (Ingraham *et al.* 2019), feature representation learning (Jing *et al.* 2021), expression referring (Yang *et al.* 2020a,b), relationship prediction (Satorras *et al.* 2021), survival gene path analysis (Liang *et al.* 2022), disease diagnosis (Xing *et al.* 2022), medical image analysis (Huang *et al.* 2022), and human action analysis (Yan *et al.* 2023). However, none of these works focus on point mutations. Our paper introduces a global local GNN based on GAT for superior representation and transformation of local mutation site features.

## 3 Methodology

Missense genetic mutations (i.e. a mistake in the DNA which results in the wrong amino acids) alter the corresponding amino acid residues in the protein sequences. The variation in physicochemical properties like charge and hydrophobicity of the residue is very likely to affect the residue-interaction network. All residues in the neighborhood of the mutation site are forced to leave the original coordinates to accommodate the modified side-chain and form another stable conformation. We used the Rosetta (Alford *et al.* 2017) FastRelax protocol to obtain the initial protein structure before and after mutation. The aim of the protein thermodynamic stability prediction task is to quantify the values of ΔΔG by learning efficient biophysical features from both the wild-type and mutant structures.

### 3.1 Global-local graph neural network

Considering that proteins exhibit a natural graph-like structure and input protein structures are inherently paired, we develop a Siamese graph neural network to extract richly structured protein features. As protein mutations arise from point mutations, the graph neural network should be capable of concentrating on the mutated site and its surrounding regions. Thus, we take the graph attention networks (GAT) (Veličković *et al.* 2018) as the backbone network of the Siamese graph neural network to extract the initial graph representations of the proteins. To learn the common knowledge of the nonmutated protein points, the upper part and the lower part of the Siamese graph network share the same weights. Formally, given a set of *N* protein node features $h=\{{\vec{h}}_{1},{\vec{h}}_{2},\ldots ,{\vec{h}}_{N}\}\in R^{N\times F}$ with the number of each node feature *F*, we first apply a shared attention mechanism to calculate the similarity ratios between a node and its neighboring nodes. For two nodes with index *i* and *j*, the importance *α_ij_* of the node *j’*s feature to that of node *i* is formulated as:


(1) $$\alpha_{ij}={\text{softmax}}_{j}(e_{ij})=\frac{\;\text{exp}(e_{ij})}{\sum_{k\in N_{i}}\;\text{exp}\;(e_{ik})},$$

(2) $$e_{ij}=\text{LeakyReLU}({\vec{a}}^{⊤}[W{\vec{h}}_{i}||W{\vec{h}}_{j}]),$$

where $W\in R^{F^{\prime }\times F}$ is a shared transformation matrix, $\vec{a}\in R^{2F^{\prime }}$ is a scoring weight vector, $N_{i}$ is the one-hot neighborhood of node *i*, || denotes the concatenation, and the softmax operation is used for normalization. Then we aggregate each node feature with its one-hot neighbors based on their similarity ratios, and adopt a multi-head concatenation operation to stabilize the training process. The aggregated feature ${\vec{h}}_{i}\prime$ of node *i* is calculated by:


(3) $${\vec{h}}_{i}^{\prime }=|{|}_{k=1}^{K}\sigma \left(\sum_{j\in N_{i}}\alpha_{ij}^{k}W^{k}{\vec{h}}_{j}\right),$$

where *K* is the number of attention heads, $\alpha_{ij}^{k}$ denotes the attention coefficients in the *k*th attention head, and $W^{k}$ denotes the transformation matrix in the *k*th head. Finally, we use the average pooling after the GAT layer to generate global protein structure representations.

Algorithm 1. Unbiased Curriculum Learning Algorithm.

**Require:** *P* = {$p_{1},\ldots ,p_{B}$}, *Y*={$y_{1},\ldots ,y_{B}$}

1: *P* denotes the prediction of the samples in mini-batch

2: *Y* denotes the label of the samples in mini-batch

3: *B* denotes the number of samples in mini-batch *D* denotes the queue to store the samples’ difficulties.

4: **for** *i *=* *1 to *B* **do**

5:  Calculate sample’s difficulty *h_i_* with Eq. (5) and store the value to queue *D*.

6: **end for**

7: Calculate the threshold *T* to filter the hard samples with Eq. (6).

8: Define the loss *L* of current mini-batch and the schedule *S* of current mini-batch with Eq. (9).

9: **for** *i *=* *1 to *B* **do**

10:  Calculate the loss *l_i_* of the sample.

11:  **if**  $h_{i}<T$  **then**

12:   $L\leftarrow L+l_{i}$

13:  **else**

14:   $L\leftarrow L+S\cdot l_{i}$

15:  **end if**

16: **end for**

17: Update model parameters with the loss *L*.

Although the above-mentioned Siamese graph attention network can represent the structural mutation process of proteins more effectively than the previous methods, it still lacks attention to local mutated nodes, which is the root cause of changes in protein topology and thermal stability. Thus, we further propose a novel and light-weighted module named Local Feature Transformation Flow (LFTF), to enhance the model’s ability to capture the local mutated node. Let *x_a_* be the local feature vector ahead of the GAT layer shaped 1×a, *x_b_* be the local feature vector behind the GAT layer shaped 1×b, the refined local feature vector after Local Feature Transform (LFT) module f(·) is represented by *y* with shape 1×b. This process is represented as:


(4) $$y=f(x_{a})+x_{b},$$

where f(·) denotes a fully connected layer with *a* input channels and *b* output channels. After that, we update the current node feature with *y* and send this feature vector into the further GAT layer and LFT layer until the last layer of the graph neural network. By taking the advantage of the LFTF module, the error during the training process could better propagate to the local node, thus our model could achieve better performance.

### 3.2 Unbiased curriculum learning

**Figure 1.**
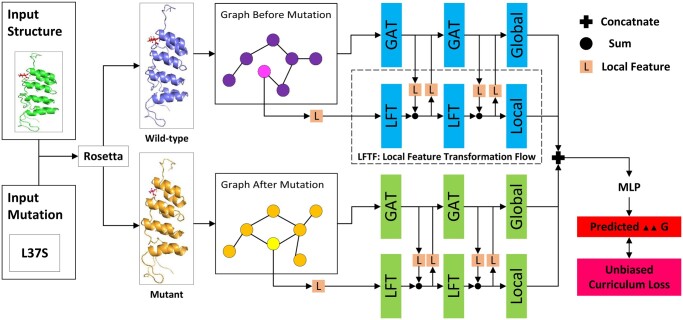
The pipeline of the proposed GLGNN-UCL: an unbiased curriculum learning-powered global-local graph neural network to predict the thermodynamic stability upon point amino acid mutation. Given an input structure and an amino acid mutation, we use Rosetta to obtain the wide-type and the mutant protein structure, which is shown in the purple and yellow part in the figure. The mutated amino acid is shown in a lighter color on the graph. The protein structure graphs are processed by a Siamese graph neural network with the Local Feature Transformation Flow (LFTF) module, to obtain its transformed feature representation. After that, we use the tailor-designed unbiased curriculum loss to train the model end to end.

To resolve the unavoidable error of the thermodynamic stability change, which is a common phenomenon in both experiments and the chemical calculation, we propose a novel unbiased curriculum learning (UCL) method to train the model end to end, which is shown in Algorithm 1. In the following three sections, we elaborate on the key concepts in UCL, which include the difficulty metric function and the curriculum scheduler (Fig. 1).

#### 3.2.1 Difficulty metric function

**Figure 2.**
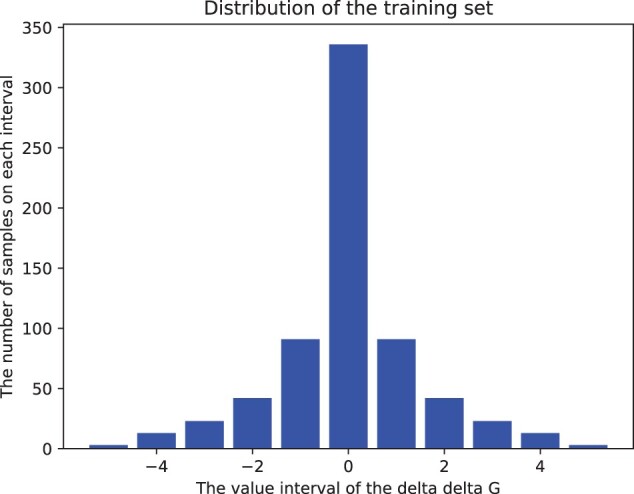
The distribution the ΔΔG values in the training set.

**Figure 3.**
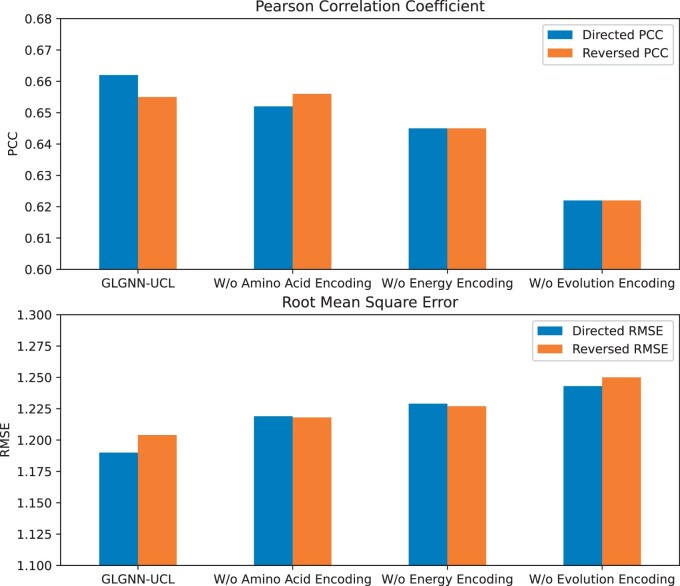
Analysis of the feature importance. “W/o” indicates “Without”.

The difficulty metric function is crucial in curriculum learning. In previous works, researchers typically use the loss of samples during training as the difficulty metric function. However, in graph regression tasks, a larger loss for a sample does not necessarily imply that the sample is harder than others, as the larger loss may result from model initialization or sample scarcity. For example, a sample with a larger ΔΔG might have a larger loss due to model initialization. To fairly select difficult samples from the current mini-batch, which contains samples with both large and small ΔΔG changes, we propose the following unbiased hardness function that eliminates the influence of ground truth values. Furthermore, to address the issue of the hardness value significantly increasing when GT (i.e. the denominator of the formula Equation 5) is close to 0, we propose the piece-wise function shown below:


(5) $$H(x)=\{\begin{array}{cc} \frac{{\left(x_{gt}-x_{\mathrm{pred}}\right)}^{2}}{\mathrm{abs}(x_{gt})} & \mathrm{abs}(x_{gt})\geq K\\ {\left(x_{gt}-x_{\mathrm{pred}}\right)}^{2} & \mathrm{abs}(x_{gt})<K \end{array},$$

where the sample is denoted by *x* with ground-truth label *x_gt_* and the predicted value is represented by *x_pred_*, the hardness of sample *x* is represented as *H*(*x*). *K* represents the piece coefficient. Given the prior knowledge that the ground truth value ΔΔG for the mutation of amino acids is typically normally distributed (see Fig. 2), we set *K* to 1. Furthermore, as illustrated in Fig. 2, the model tends to overfit on samples with ground-truth values around 0, as they constitute a large portion of the entire dataset. In this situation, samples with larger ground truth values are likely to be assigned a larger loss. However, the difficulty of samples may be influenced by factors such as intrinsic topological structure and node features, in addition to the ground truth value. Thus, our unbiased design addresses this issue from another perspective. Based on the above unbiased measurement function, we design an adaptive threshold to assess each sample’s difficulty according to the average and deviation of sample difficulties in a mini-batch, which is formulated as follows (Fig. 3):


(6) $$T_{\mathrm{cur}}=h_{\mathrm{avg}}+\alpha \cdot h_{\mathrm{std}},$$

where *α* is a hyper-parameter for hard sample mining which is set to 1 by default. The higher *α* is, the fewer samples are regarded as the hard sample. The *h_avg_* and *h_std_* denote the averaged difficulty and the standard deviations of difficulty in the current batch, which are defined below:


(7) $$h_{\mathrm{avg}}=\frac{1}{N}\sum_{n=1}^{n=N}H(x_{n}),$$

(8) $$h_{\mathrm{std}}=\frac{1}{N}\sum_{n=1}^{n=N}(H(x_{n})-h_{\mathrm{avg}}{)}^{2},$$

where the number of samples contained in current mini-batch is represented by *B*.

#### 3.2.2 Scheduler design

After identifying difficult samples using the aforementioned methods, it is necessary to design a scheduler function for noisy samples to adaptively adjust their importance. The question arises: should we learn from easy to hard samples (i.e. weight the hard samples from 0 to 1) or from hard to easy (i.e. weight the hard samples from 1 to 0)? Training the model from easy to hard may lead to overfitting on noisy samples, even though the model is not affected by difficult samples initially. On the other hand, learning from hard to easy, an anti-curriculum paradigm, offers several advantages: (i) The model can extract more topological information from hard samples on the amino acid graph in the early stages, thereby avoiding overfitting on noisy outliers in the final stage. (ii) During the initial training rounds, the model may not accurately identify difficult samples. This uncertainty could cause the model to misclassify easy samples as hard samples. As a result, learning from easy to hard may lead to the inadvertent discarding of misjudged “hard samples” (which are actually easy samples) at the beginning, adversely affecting the model’s generalization ability. Therefore, we choose to learn from hard to easy. Let *E* be the number of training epochs, *e* denotes the current epoch, and the anti-curriculum schedule is defined as:


(9) $$S(e)=1-\frac{e}{E}.$$

### 3.3 Loss function

By taking the above-mentioned curriculum paradigm into account, let *h_i_* be difficulty of *i*th sample in a mini-batch that is obtained by Equation 5., *x_i_* and *y_i_* denotes the predicted value and ground truth value of the ΔΔG for sample *i*, the re-weighted loss $l\prime_{i}$ is defined as:


(10) $$l\prime_{i}=\{\begin{array}{cc} {\left\|x_{i}-y_{i}\right\|}_{2} & h_{i}\leq T_{\mathrm{cur}}\\ S*{\left\|x_{i}-y_{i}\right\|}_{2} & h_{i}>T_{\mathrm{cur}} \end{array}.$$

The final loss with respect to a mini-batch is defined as:


(11) $$L=\frac{1}{N}\sum_{i=1}^{N}l\prime_{i}.$$

## 4 Experiments

### 4.1 Dataset

The training dataset in this study is derived from FireprotDB (Stourac *et al.* 2021), which contains 2518 samples upon single-point mutation after removing replicated mutations and the homologous proteins against the test data (BLAST *P*-value ≥ 0.001) (Li *et al.* 2020). We perform the 5-fold cross-validation on the training set, which means using 2014 samples for training and selecting the best performing model on the remaining 504 samples in the validation set. The Ssym dataset (Pucci *et al.* 2018) that contains 684 mutated samples is used for testing. PDBrenum Faezov and Dunbrack Jr (2021) was used to convert the mutation positions in the database to those in the PDB structures. The procedure to represent the proteins in the form of the graph are summarized as follow. If the distance between the alpha C of the amino acids <5 Å, we add a connecting edge between two amino acid nodes. For the feature of the nodes (i.e. amino acid residues) in the graph, they are obtained from the following three categories: (i) Amino acid encoding, including 5D representation from skip-gram model (Lv *et al.* 2021), 7D one-hot vector according to the amino acid classification, 8D vector summarizing several basic biophysical properties of a single residue. (ii) Energy encoding, 20D representation from Rosetta scoring functions (Alford *et al.* 2017), including both physics-based (Van der Waals interactions, solvation, hydrogen bonds) and knowledge-based energy terms (protein backbone, side-chains, torsions). (iii) Evolutionary encoding, 20D representation derived from multiple sequence alignment against the Uniclust_30 database (Mirdita *et al.* 2017) by *hhblits* (Remmert *et al.* 2011). To sum up, we obtain a 60D feature to encode each node in the graph. We obtain the edge information of the graph from the interaction of amino acid residues.

### 4.2 Implementation and metric

All the models are trained with NVIDIA RTX 3090 GPU with 24 GB memory. The framework is implemented in PyTorch 1.10.1 and PyTorch Geometric 2.0.2 (Fey and Lenssen 2019) and CUDA 10.6. AdamW is applied to optimize the model. We train the models at a learning rate of 0.002, batch size at 256, training epoch at 50, and weight decay at 0.001. It is worth noting that as PyTorch Geometric does not guarantee reproduction, the results of the SiamGNN methods and the LIR-based methods are obtained by averaging the result of five independent experiments with 5-fold cross-validation. We follow the previous works (Li *et al.* 2020, Benevenuta *et al.* 2021, Pancotti *et al.* 2022) to use the Root Mean Square Error (RMSE), Pearson Correlation Coefficient (PCC), and Anti-symmetric score as the metric for model evaluation.

### 4.3 Comparison with state-of-the-art methods

We evaluate our GLGNN model in three ways: (i) We compare it with previous top-performing models to demonstrate its effectiveness. (ii) We test our Siamese graph network model with three popular graph neural network backbones and our GLGNN backbone. (iii) We conduct experiments on GLGNN-UCL and other advanced methods addressing noisy samples in regression tasks. The test set is divided into “Direct” (mutations in the natural protein) and “Reverse” (mutations prior to the natural protein) to highlight the proposed methods’ impact (Table 1).

**Table 1.** Details of dataset used in our experiments, including dataset names, types, and number of mutated proteins.

Dataset	FDB	FDB	Ssym	P53
Type	Training	Validation	Testing	Testing
Size	2014	504	684	168

The results comparing various network structures are shown in the upper part of Tables 2 and 3. By utilizing Siamese graph representation, most graph-based methods surpass previous neural network-based methods. GAT outperforms other methods because it can focus on the mutated site. More importantly, our GLGNN, which uses a tailored local feature transformation flow, can better learn local features. As a result, GLGNN not only outperforms previous neural network-based methods but also significantly surpasses other graph representation methods.

**Table 2.** Comparison with state-of-the-art methods on Ssym benchmark.^a^

Setting	Methods	RMSE			PCC			Anti-symmetric	
		Direct	Reverse	Average	Direct	Reverse	Average	*r*_*div−rev*_	*δ*
Previous SOTA	SDM (Pandurangan *et al.* 2017)	1.74	2.28	2.01	0.51	0.32	0.42	−0.75	−0.32
	PopMusicSym (Pucci *et al.* 2018)	1.58	1.62	1.60	0.48	0.48	0.48	−0.77	−0.06
	DDGun3D (Montanucci *et al.* 2019)	1.42	1.46	1.44	0.56	0.53	0.55	−0.99	−0.04
	ThermoNet (Li *et al.* 2020)	1.56	1.55	1.56	0.47	0.47	0.47	−0.96	−0.01
	ACDC-NN (Benevenuta *et al.* 2021)	1.45	1.45	1.45	0.57	0.57	0.57	−0.98	−0.05
	KORPM (Hernández *et al.* 2023)	1.28	1.38	1.33	0.57	0.49	0.53	−0.88	−0.15
**SiamGNN**	GraphSAGE (Hamilton *et al.* 2017)	1.49	1.48	1.48±0.02	0.38	0.39	0.39±0.02	−0.98	−0.02
	GAT (Veličković *et al.* 2018)	1.34	1.34	1.34±0.01	0.55	0.55	0.55±0.01	−0.99	−0.02
	GIN (Xu *et al.* 2019)	1.40	1.41	1.40±0.01	0.48	0.47	0.47±0.02	−0.98	−0.02
	**GLGNN**	**1.23**	**1.23**	**1.23±0.01**	**0.63**	**0.63**	**0.63±0.01**	−0.99	−0.03
LIR	**GLGNN**+SL (Castells *et al.* 2020)	1.22	1.23	1.22±0.01	0.64	0.64	0.64±0.01	−0.99	−0.02
	**GLGNN**+DIR (Yang *et al.* 2021)	1.23	1.24	1.23±0.01	0.64	0.65	0.64±0.01	−0.99	−0.02
	**GLGNN+UCL**	**1.21**	**1.20**	**1.20±0.02**	**0.66**	**0.66**	**0.66±0.02**	−0.99	−0.02
	**GLGNN+UCL** ^b^	**1.25**	**1.24**	**1.24±0.02**	**0.62**	**0.63**	**0.63±0.02**	−0.99	−0.02

a In SiamGNN, we compare different backbones based on the SiamGNN framework. In LIR (learning with imbalance regression), we compare the proposed UCL with SL (NeurIPS’20) and DIR (ICML’21) based on our GLGNN.

b Separately averaging the results of five folds. Unique best results and our methods are marked in **bold**.

**Table 3.** Comparison with state-of-the-art methods on the P53 benchmark.^a^

Setting	Methods	RMSE			PCC			Anti-symmetric	
		Direct	Reverse	Average	Direct	Reverse	Average	*r*_*div−rev*_	*δ*
NN-based	ThermoNet (Li *et al.* 2020)	2.01	1.92	1.96	0.45	0.56	0.50	−0.97	−0.02
	ACDC-NN (Benevenuta *et al.* 2021)	1.67	1.72	1.70	**0.62**	**0.61**	**0.61**	−0.99	−0.01
**SiamGNN**	GraphSAGE (Hamilton *et al.* 2017)	1.74	1.74	1.74±0.02	0.44	0.44	0.44±0.01	−0.98	−0.02
	GAT (Veličković *et al.* 2018)	1.77	1.77	1.77±0.01	0.54	0.55	0.54±0.01	−0.99	−0.02
	GIN (Xu *et al.* 2019)	1.76	1.77	1.76±0.02	0.49	0.48	0.48±0.02	−0.99	−0.02
	**GLGNN**	**1.57**	**1.58**	**1.57±0.01**	**0.61**	**0.60**	**0.61±0.01**	−0.99	−0.02
LIR	**GLGNN**+SL (Castells *et al.* 2020)	1.57	1.58	1.57±0.01	0.59	0.59	0.59±0.02	−0.99	−0.03
	**GLGNN**+DIR (Yang *et al.* 2021)	1.61	1.61	1.61±0.02	0.59	0.60	0.59±0.02	−0.99	−0.02
	**GLGNN+UCL**	**1.55**	**1.54**	**1.55±0.02**	**0.65**	**0.65**	**0.65±0.02**	−0.99	−0.02
	**GLGNN+UCL** ^b^	**1.60**	**1.59**	**1.59±0.02**	0.60	0.60	0.60±0.02	−0.99	−0.02

a In SiamGNN, we compare different backbones based on the SiamGNN framework. In LIR (learning with imbalance regression), we compare the proposed UCL with SL (NeurIPS’20) and DIR (ICML’21) based on our GLGNN.

b Separately averaging the results of five folds. Unique best results and our methods are marked in **bold**.

Regarding learning methods aimed at handling noisy samples in regression tasks, we compare the proposed GLGNN-UCL with two advanced learning methods in the LIR (Learning with Imbalanced Regression) part of Tables 2 and 3. We carefully adjust their hyperparameters to ensure optimal performance. On the Ssym benchmark, “SL” and “DIR” slightly improve the model’s performance. However, on the P53 benchmark, “DIR” and “SL” fail to enhance the model’s performance. In contrast, the proposed “UCL” boosts performance on both Ssym and P53 benchmarks. The reason behind this might be that “DIR” mainly focuses on the imbalance issue in regression tasks and does not sufficiently consider noisy samples. The rebalancing strategy in “DIR” further increases noise in the training data. As for “SL”, it overlooks the intrinsic label value bias. Consequently, the proposed “UCL” approach achieves state-of-the-art results among previous works by reducing the influence of samples with different ground truth values.

### 4.4 Ablation study

**Ablation on the model structure.**  Table 4 presents the ablation study focusing on the model structure. In this table, “Global” refers to the vanilla graph representation method (GAT). “Global+LFA (local feature aware)” represents training the model under the supervision of both global features and local mutated site features by concatenating them into a single feature vector. “Global+LFTF (local feature transform flow)” denotes training the model with the local feature transform flow. The results show that incorporating local information improves the accuracy of protein thermodynamic stability prediction, as protein structure mutations originate from mutated amino acids (i.e. local mutated nodes). Moreover, the transform layer effectively enhances performance, linearly transforming the local feature with only 0.05 MB parameters.

**Table 4.** Ablation study of the model structure based on the Ssym test set.^a^

Metric	Global	Global+LFA	Global+LFTF
RMSE	1.34±0.02	1.27±0.01	1.23±0.01
PCC	0.55±0.02	0.60±0.01	0.63±0.01

a “LFA” denotes the “local feature aware” module. “LFTF” denotes the “local feature transformation” module.

**Ablation on the Unbiased Anti-curriculum method.** In Table 5, we assess three curriculum approaches. M1 is a biased method using loss as a difficulty measure. M2 uses a proposed unbiased difficulty metric and an easy-to-hard scheduler (i.e. S=e/E). M3 is an unbiased anti-curriculum method with a hard-to-easy schedule. From Table 5, both proposed M1 and M3 outperform the baseline, indicating that the “Unbiased” operation effectively distinguishes hard samples. The results between M1 and M3 support the assumption in the “scheduler design” section (Section 3.2.2). All the results surpass the baseline, demonstrating that down-weighting samples with noise is effective.

**Table 5.** Ablation study of the proposed unbiased curriculum learning method.

Method	Unbiased	Anti-curri	Metric	
			RMSE	PCC
Baseline			1.23±0.01	0.63±0.01
M1	*✓*		1.22±0.01	0.64±0.01
M2		*✓*	1.23±0.01	0.64±0.01
M3	*✓*	*✓*	1.20±0.01	0.66±0.01

**Sensitivity analysis on the hard sample metric function.** For noisy sample mining, we provide a sensitivity analysis of the hyperparameter *α* in Equation 6, as shown in Table 6. The results demonstrate that all the *α* values significantly outperform the baseline method, indicating the effectiveness of our noisy sample detection algorithm.

**Table 6.** Sensitivity analysis on the hard sample select function.

*α*	Baseline	0.5	1	2
RMSE	1.23±0.01	1.20±0.01	1.20±0.01	1.21±0.01
PCC	0.63±0.01	0.66±0.01	0.65±0.01	0.65±0.01

## 5 Discussion and conclusion

The analysis of feature importance is shown in Fig. 2. The GLGNN-UCL model, with all features included, surpasses other models, providing superior Directed and Reversed PCC and lower RMSE values, indicating a strong correlation with low error rates. Models lacking amino acid or energy encoding show slightly reduced PCC and slightly increased RMSE, indicating minor losses in accuracy. The worst-performing model lacks evolutionary encoding, having the lowest PCC and highest RMSE values, emphasizing the vital role of evolutionary encoding. We think the reason might be that evolutionary encoding aids in understanding the intricate links between protein sequences, structures, and functions, which are key to stability predictions. Moreover, if a mutation occurs in an evolutionarily conserved region, it’s likely to have a significant impact on protein stability, which might also be the reason why the evolutional feature could boost the performance. Overall, these results highlight that each feature encoding uniquely contributes to GLGNN’s performance, with evolutionary encoding being particularly crucial. This aligns with the idea that protein behavior, a complex phenomenon, is influenced by a mix of factors, necessitating a diverse feature set in machine learning models predicting protein behaviors.

Our study focuses on single-point mutations due to their significant impact, using the GLGNN model. Although this model could hypothetically predict the effects of multiple mutations by treating each as an individual single-point mutation, we discourage this due to potential complex, nonlinear interactions between mutation sites. We’re developing a new model to accurately predict both single and multiple-point mutations for a more comprehensive mutation impact prediction tool. For further wet-lab experiment, we outline a plan which is available in the appendix.

In this study, we present GLGNN-UCL, a graph regression method incorporating curriculum learning to address the problem of protein thermodynamic stability prediction. We first introduce a custom-designed global-local graph network to predict the thermodynamic change in proteins upon amino acid mutation. Subsequently, we propose an unbiased curriculum learning paradigm to handle noisy samples during training by controlling the weight of these samples. Comprehensive experimental results on a widely used benchmark confirm the superior performance of our approach. It not only outperforms advanced protein stability prediction methods based on neural networks or graph neural networks but also demonstrates superiority among state-of-the-art learning methods for regression prediction tasks. Notably, our local feature transformation module requires only 0.05 MB parameters but boosts performance by approximately 4%. More interestingly, the custom-designed UCL module enhances performance by 3% without any increase in parameters.

In addition, our work not only addresses the gap in protein thermodynamic stability prediction but also pioneers a way to handle noisy samples in the field of graph regression. Furthermore, we contribute a benchmark for evaluating graph neural networks on the PTSP task. Future work will involve delving deeper into the curriculum paradigm by exploring tailor-designed schedulers and validating the performance of our algorithm through wet laboratory experiments.
